# Real-Time Fatigue Monitoring Using sEMG and HRV Sensors for Industrial Operators Under Swing Conditions

**DOI:** 10.3390/s26154761

**Published:** 2026-07-27

**Authors:** Jichong Lei, Cannan Yi, Hong Hu, Tao Qing, Yinjuan Kang, Yuanhao Mi, Zhao Zheng, Kun Xu, Hongliang Xu

**Affiliations:** 1 School of Safety and Management Engineering, Hunan Institute of Technology, Hengyang 421002, China; 2 School of Computer Science and Engineering, Central South Unviersity, Changsha 410083, China; 3 School of Computer Science and Engineering, Hunan Institute of Technology, Hengyang 421002, China

**Keywords:** real-time monitoring, sEMG and HRV sensors, operational fatigue, multimodal fusion, industrial operator safety

## Abstract

Real-time monitoring of operator fatigue is critical for ensuring operational safety and reliability in dynamic industrial environments, especially under swing conditions such as offshore floating nuclear power platforms. This study proposes a multimodal fatigue monitoring framework based on surface electromyography (sEMG) and heart rate variability (HRV) sensors for real-time fatigue recognition. Experiments were conducted on a six-degree-of-freedom motion platform with three swing levels, involving 23 participants performing simulated emergency operation tasks. Four machine learning models (Naive Bayes, K-Nearest Neighbor, Multilayer Perceptron, and Random Forest) were employed for fatigue state classification. The results show that the Random Forest model achieves the best performance, with an overall accuracy of 98.2%, 100% true positive rate for the normal state and fatigue, and 66.7% true precision for severe fatigue. The proposed multimodal fusion method effectively suppresses motion artifacts and improves recognition robustness under swing interference. Rigorous subject-level stratified cross-validation eliminates sample leakage risks; bootstrap confidence intervals and pairwise significance tests statistically verify model performance differences; class imbalance mitigation strategies are deployed to quantify uncertainty for the scarce severe-fatigue category; literature-supported Borg CR-10 grading thresholds are validated via retrospective cutoff sensitivity analysis to guarantee reliable fatigue labeling. This sensor-based intelligent monitoring system provides a reliable solution for real-time fatigue detection of operators in dynamic digital industrial scenarios, supporting accident prevention and sustainable operation of high-risk industrial systems.

## 1. Introduction

Driven by the advancement of Industry 4.0 and the proliferation of intelligent manufacturing, digital industrial systems, marked by high degrees of automation and intelligence, have seen widespread adoption across sectors such as maritime engineering, aerospace, and ocean technology. These systems have significantly enhanced operational efficiency and managerial effectiveness [1,2,3,4,5]. However, dynamic swing environments prevalent in these fields, such as ship roll and pitch in turbulent seas or the attitude variations of aerospace vehicles in unstable atmospheric conditions, impose substantial cognitive and physical burdens on operators, often resulting in operational fatigue [6,7,8]. This fatigue not only delays response times and impairs decision-making but also increases the likelihood of severe safety incidents, posing serious threats to both human lives and material assets. Consequently, accurately detecting operator fatigue under such swing conditions has become a pressing issue in the domain of industrial safety [9,10].

Historically, fatigue recognition methods have relied heavily on subjective assessments and single-modality analyses of physiological or behavioral features [11,12,13]. Subjective tools, such as self-report fatigue scales, are influenced by individual perception and communication ability, introducing considerable bias and latency in detection [14,15]. Meanwhile, objective methods utilizing unimodal physiological signals (e.g., EEG, PPG) or behavioral indicators (e.g., facial expressions, body posture) offer more standardized assessments but often lack robustness in swing-intensive settings due to their susceptibility to environmental noise and motion artifacts [16,17,18].

Recent advancements in sensor technologies and artificial intelligence have unlocked new possibilities for multimodal data fusion and intelligent fatigue detection. By integrating data from various sources, multimodal approaches can extract fatigue-related patterns from diverse perspectives, mitigating the limitations of single-modality techniques and improving the accuracy and reliability of recognition outcomes [19,20,21,22]. Despite this progress, most existing studies have focused on stable, conventional operational environments, with limited research addressing multimodal data acquisition and fatigue recognition in swing conditions.

The swing-prone environments of digital industrial systems introduce distinctive challenges [23,24,25]. Compared with static monitoring tasks, 6-DOF swing environments force operators to continuously maintain body stability through isometric contraction of core and limb muscles, which significantly changes muscle activation patterns [26]. Biomechanically, continuous anti-sway control increases muscle co-activation, reduces sEMG median frequency (MF), and amplifies motion artifacts in the time–frequency domain. Physiologically, long-term swing stimulation activates the sympathetic nervous system, suppresses parasympathetic activity, increases HRV low-frequency (LF) components, decreases high-frequency (HF) components, and significantly elevates the LF/HF ratio, a classic biomarker reflecting autonomic imbalance under combined postural and cognitive load [26]. Continuous vestibular perturbation and sustained isometric muscle contraction jointly shift cardiac autonomic regulation toward sympathetic dominance, which has been validated in human factor studies on offshore dynamic operation environments [27].

The selection of sEMG and HRV as core fatigue monitoring modalities is not arbitrary, and their physiological mechanisms for fatigue assessment are well-established in the existing literature.

For sEMG, reduction in median frequency (MF) is a gold-standard marker of peripheral skeletal muscle fatigue during sustained contraction. Three core physiological mechanisms jointly induce this downward spectral shift: first, the “muscle wisdom” effect, where metabo-reflex triggered by muscle chemo- and nociceptors reduces motor unit discharge frequency to conserve energy; second, slowed action potential propagation velocity along fatigued muscle fibers; third, elevated synchronization of motor unit firing sequences [28,29]. These three synergistic phenomena allow muscles to maintain target contraction force under accumulated metabolite deficits, making MF extracted from multi-channel sEMG reliable for quantifying localized anti-sway muscle fatigue in swing conditions.

For HRV, frequency domain indices are sensitive to combined physical fatigue and vestibular-induced mental stress, capturing central autonomic responses that sEMG cannot reflect independently. Under continuous dynamic posture maintenance, cumulative physical burden and persistent swing stimulation jointly raise sympathetic activity and inhibit parasympathetic modulation, which can be quantitatively characterized via LF, HF and LF/HF ratio [30]. Single-modality detection has obvious defects: sEMG signals are severely contaminated by swing motion artifacts and cannot reflect overall central nervous fatigue; HRV only characterizes systemic autonomic stress without capturing localized muscle exhaustion. Multimodal fusion of sEMG and HRV compensates their complementary physiological information, which is the core design logic of the proposed monitoring framework.

These changes are not obvious in static environments and lead to distinct fatigue characteristics under swing conditions. These complexities underscore the urgent need for specialized multimodal intelligent recognition methods that can accommodate the unique characteristics of such environments and enable real-time, accurate monitoring of operator fatigue.

This study addresses these challenges by systematically exploring the multimodal manifestations of operator fatigue in digital industrial systems under swing conditions. It aims to design an efficient multimodal data acquisition platform, develop advanced data fusion strategies and intelligent recognition algorithms, and ultimately propose a high-accuracy, high-reliability framework for detecting fatigue states. The anticipated outcomes will not only deepen theoretical understanding in the field of fatigue detection but also contribute novel insights and methodologies to ergonomics under extreme operational contexts. From a practical standpoint, the proposed approach can help enterprises monitor fatigue levels in real-time and implement targeted interventions, thereby reducing the incidence of fatigue-related accidents and enhancing overall system safety and resilience. This research holds substantial theoretical significance and strong potential for engineering application. Although multimodal physiological fatigue assessment has been widely studied in static operational environments, the true novelty of this study lies in the fusion of sEMG and HRV for real-time fatigue monitoring under 6-DOF swing conditions, which is rarely addressed in the existing literature. Swing-induced motion artifacts and continuous postural adjustment significantly change muscle activation and autonomic nervous responses, making traditional static multimodal methods ineffective. The proposed multimodal fusion framework can suppress motion interference, extract complementary fatigue features from sEMG and HRV, and achieve robust recognition in dynamic industrial environments such as offshore floating nuclear power platforms. This work strictly unifies subject-independent stratified Leave-One-Subject-Out Cross-Validation (LOSO-CV) as the core evaluation standard and clarifies the auxiliary positioning of the 9:1 subject group split to eliminate data leakage risks. Statistical bootstrap confidence intervals and significance tests are supplemented to reliably validate the superiority of random forest, and imbalance mitigation strategies are applied to reduce bias caused by the low proportion of severe-fatigue samples.

## 2. Methodology and Principles

### 2.1. Naive Bayes

The Naive Bayes algorithm is a commonly used classification method in data mining [31,32,33]. Based on statistical data, it assumes that all features are mutually independent and calculates the probability that a test sample belongs to each class, assigning it to the class with the highest probability. Given a training set B containing N samples and m classes (denoted as $A=\left\{A_{1},A_{2},\ldots ,A_{m}\right\}$), let $N_{A_{j}},j=1,2,\ldots ,m.$ represent the number of samples in class j, then we have $\sum_{j=1}^{m}N_{A_{j}}=N$. Suppose each sample contains n attributes, denoted as $C_{1},C_{2},\ldots ,C_{n}$. The *k*-*th* sample $x_{k}$ is represented as $x_{k}=\left(x_{k_{1}},x_{k_{2}},\ldots ,x_{k_{n}}\right),k=1,2,\ldots ,N$, where $x_{k_{i}}$ denotes the value of the *k*-*th* sample on attribute $C_{i}$. Given a test sample $h=\left(h_{1},h_{2},\ldots ,h_{n}\right)$, where $h_{i}$ represents the value of h on attribute $C_{i}$, the conditional probability that the test sample h belongs to class $A_{j}$ can be computed using the Naive Bayes algorithm as follows:

(1) $$P\left(\left.A_{j}\right|h\right)=\frac{P\left(A_{j}\right)}{P\left(h\right)}\prod_{i=1}^{n}P\left(\left.A_{j}\right|h_{j}\right)\;\;\;\;\;\;i=1,2,\ldots ,n\;\;\;\;\;\;j=1,2,\ldots ,m.$$

(2) $$P\left(A_{j}\right)=\frac{N_{A_{j}}}{N}$$

Here, $P\left(A_{j}\right)$ denotes the prior probability of class $A_{j}$; $P\left(\left.A_{j}\right|h\right)$ represents the probability that the test sample h belongs to class $A_{j}$; $P\left(\left.h_{j}\right|A_{j}\right)$ indicates the conditional probability that the test sample h takes the value $h_{i}$ on attribute $C_{i}$, given that it belongs to class $A_{j}$.

### 2.2. K-Nearest Neighbor Algorithm

The K-Nearest Neighbor (KNN) algorithm, proposed by Cover and Hart in 1968, is a fundamental classification method [34,35]. Its working principle is simple and intuitive: given a labeled training dataset, the algorithm classifies an input sample based on the majority class of its K nearest neighbors in the training set, determined by a specified distance metric. Specifically, for a test sample, the algorithm calculates the distance between this sample and each sample in the training set, selects the K samples with the smallest distances, and assigns the test sample to the class most frequently represented among these K neighbors.

Assuming a dataset containing several sEMG and HRV signal samples $T=\left\{\left(X_{1},Y_{1}\right),\left(X_{2},Y_{2}\right),\ldots ,\left(X_{N},Y_{N}\right)\right\}$ with corresponding fatigue category labels, let $X=\left[x_{1},x_{2}\right]$ denote the feature vector of a detection signal, where $x_{1}$ represents sEMG features and $x_{2}$ represents HRV features. The classification process can proceed as follows.

Step 1: Calculate the Euclidean distance between the detection signal feature vector X and each sample in the dataset. The Euclidean distance between X and a training sample $x_{i}$ is given by

(3) $$L\left(X,X_{i}\right)={\left(\sum_{p=1}^{2}{\left(X^{p}-X_{i}^{p}\right)}^{2}\right)}^{\frac{1}{2}}$$

In the equation, $X^{p}$ denotes the *p*-*th* feature value of the measured (test) sample, and $X_{i}^{p}$ denotes the *p*-*th* feature value of the *i*-*th* sample in the dataset.

Step 2: Count the categories of the K nearest sample points. According to the majority voting principle, the category that appears most frequently is assigned as the category of the test sample. Assuming that there are C designed fatigue categories, the predicted category of the test sample is determined as follows:

(4) $$C\left(X\right)=\underset{C_{i}}{\max}\;k_{i}$$

In this equation, $k_{i}$ refers to the total number of samples belonging to category $C_{i}$ within the $C_{i}$ nearest neighbors of the test sample X. The proximity of neighbors is quantified by Euclidean distance. $C_{i}$ denotes the label of the *i*-*th* fatigue category. The test sample is assigned to the category with the largest $k_{i}$ following the majority voting rule of KNN.

### 2.3. Multilayer Perceptron

**Figure 1:**
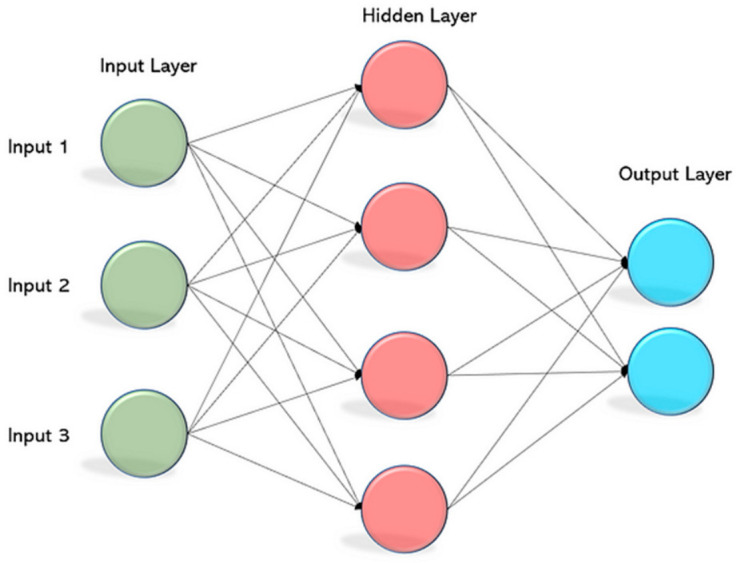
Structure diagram of Multi-Layer Perceptron.

Multilayer Perceptron (MLP), proposed by Rosenblatt, is an artificial neural network widely applied across various fields [36,37,38]. It typically consists of an input layer and an output layer, with multiple hidden layers in between. The MLP architecture is illustrated in Figure 1.

A set of synaptic connections with heterogeneous weights constitutes an MLP. Within each layer, the connections are combined via weighted summation plus bias, and then passed through an activation function to obtain the output. Assuming there are a total of m hidden layers, let w represent the weight of the *k*-*th* neuron in the *i*-*th* layer, and $x_{k_{i}}$ denote the input to the *k*-*th* neuron in the *i*-*th* layer. Since the MLP employs a fully connected architecture between layers, when the input layer contains n neurons, the output $x_{k_{2}}$ of the neurons in the first hidden layer is given by

(5) $$x_{k_{2}}=f\left(\sum_{k=1}^{n}w_{k_{1}}x_{k_{1}}+b_{k_{1}}\right)$$

In the equation, $b_{k_{1}}$ represents the bias term of the *k*_1_-*th* neuron, and $f\left(\cdot \right)$ denotes the activation function. The input to the next hidden layer is the output of the previous hidden layer, based on which the output of any neuron in other hidden layers can be further obtained as follows:

(6) $$x_{k_{i}}=f\left(\sum_{k=1}^{n}w_{k_{\left(i-1\right)}}x_{k_{\left(i-1\right)}}+b_{k_{\left(i-1\right)}}\right),i=2,3,\ldots ,m$$

The final output of the model is obtained by performing a weighted summation of the outputs y from the last hidden layer, as expressed by

(7) $$y=f\left(\sum_{k=1}^{n}w_{k_{m}}x_{k_{m}}+b_{k_{m}}\right)$$

For activation functions f, the main types include the sigmoid( ) function, tanh( ) function, ReLU( ) function, and Softmax( ) function. In this study, the MLP model adopts the sigmoid( ) function as the activation function, specifically

(8) $$f\left(z\right)=\frac{1}{1+e^{-z}}$$

Its derivative is

(9) $$f^{\prime }\left(z\right)=f\left(z\right)\left(1-f\left(z\right)\right)$$

### 2.4. Random Forest

Random Forest (RF) is a machine learning method used for classification or regression tasks [39,40]. It enhances overall model accuracy and generalization performance by aggregating an ensemble of decision trees, with its core principle being the integration of multiple weak learners to form a strong classifier. In practical applications, datasets often contain numerous feature variables, making it particularly important to identify and retain only those that significantly impact the outcome to reduce model complexity. Compared to methods such as principal component analysis and regression analysis, Random Forest can handle many predictor variables simultaneously and provides an importance measure for each feature.

**Figure 2:**
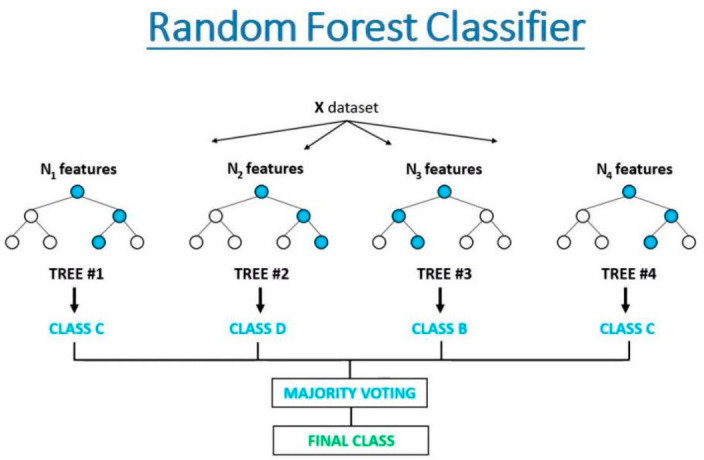
Schematic Representation of the Random Forest Architecture.

In Random Forest regression, the bagging technique is employed: bootstrap or subsample data is randomly drawn from the training set for the construction of each decision tree. Although each random sample reflects the same data-generating process, the stochastic variations cause each tree to depend heavily on its specific training data, resulting in significant diversity among the trees. Furthermore, the trees built in Random Forest are typically large and incorporate combinations of various predictor variables. By aggregating the predictions from these diverse trees, the method leverages the inherent instability of regression trees to perform averaging, thereby making the final prediction more accurate. This ensemble strategy generally achieves superior predictive accuracy compared to any single classification or regression tree. An illustrative example of this concept is provided in Figure 2.

The Random Forest (RF) algorithm presents numerous strengths: it is conceptually straightforward, easy to implement, computationally efficient, and particularly well-suited for tasks involving high-dimensional feature spaces and redundant information. Owing to these attributes, RF has demonstrated outstanding predictive performance across a diverse range of real-world applications. Since its introduction in 2001, it has been extensively utilized in domains such as ecology, environmental monitoring, and land-use classification. This algorithm is not only fast and accurate but also capable of evaluating variable importance, capturing complex interactions among predictors, and reducing the dependence on large training datasets.

Within an RF model, decision-making at each node is guided by the optimal split chosen from a randomly selected subset of predictor variables. This randomness in feature selection enhances both the robustness and generalization capability of the model. Furthermore, RF employs bootstrap sampling, resampling the original dataset with replacement to construct individual trees, a process central to the bagging approach. This strategy increases diversity among trees and mitigates the risk of overfitting, thereby improving overall model accuracy. Other notable advantages of RF include its adaptability in determining the number of trees based on user requirements, its rapid response time, and its resilience to overfitting. In most cases, the construction of each decision tree within the forest relies on the Gini index as the criterion for split selection.

The Gini index is used to evaluate the impurity of a node, aiming to achieve the highest purity (i.e., homogeneity) in each child node after a split. The purer the node, the lower the Gini index, indicating reduced uncertainty. For a standard decision tree with K classes, if the probability that a sample belongs to class k is $p_{k}$, then the Gini index for that node is defined as

(10) $$Gini\left(p\right)=\sum_{k=1}^{K}p_{k}\left(1-p_{k}\right)=1-\sum_{k=1}^{K}p_{k}^{2}$$

The larger the Gini index, the greater the uncertainty; the smaller the Gini index, the lower the uncertainty and the more thorough the data split. For a CART (Classification and Regression Tree), which is a binary decision tree, the Gini index for a node can be expressed as

(11) $$Gini\left(p\right)=2p\left(1-p\right)$$

In this formula, the term p represents the probability of a specific class label occurring. Typically, this probability indicates the likelihood that a given data sample belongs to a particular class.

## 3. Experimental Design

### 3.1. Experimental Background and Conditions

**Figure 3:**
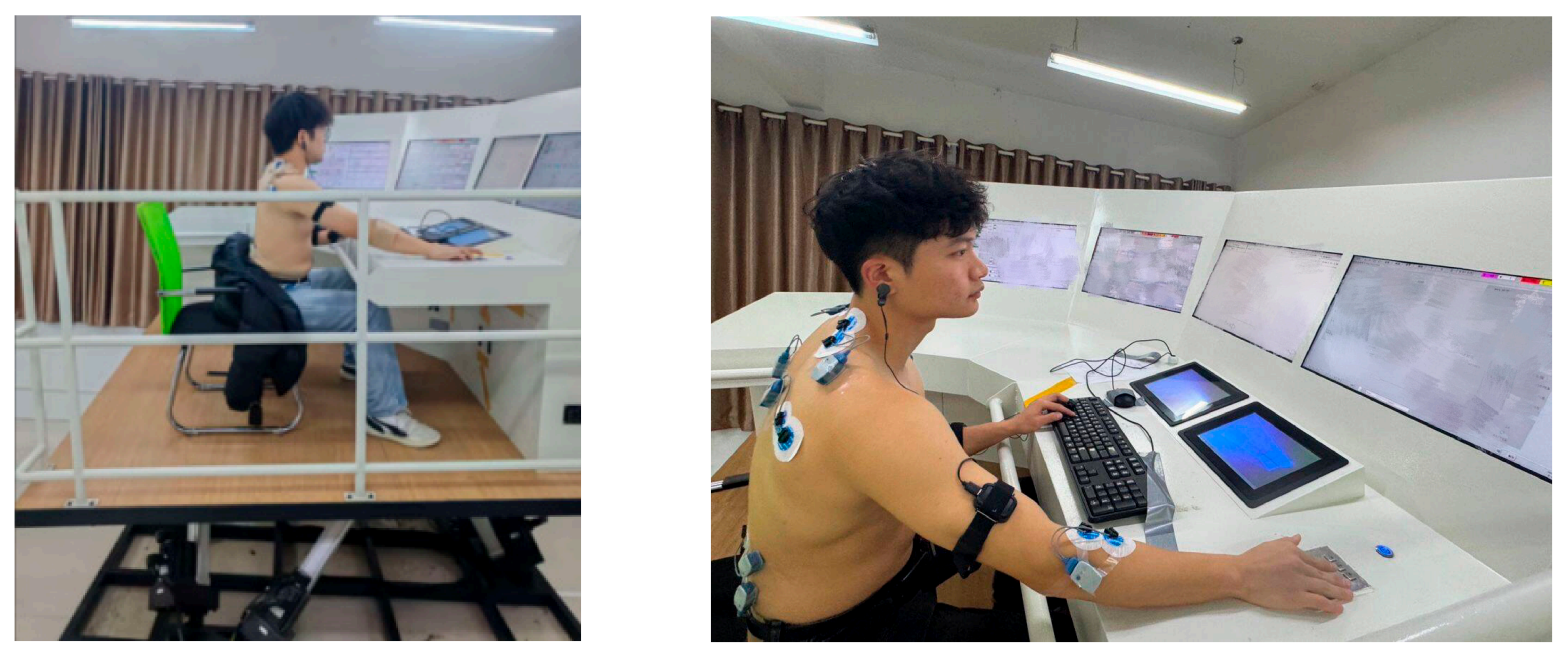
Operator performing tasks under vibrating conditions.

As a representative digital industrial system, nuclear power operations impose a significant workload on operators under accident conditions, often leading to the accumulation of operational fatigue. In response, the academic community has initiated studies on operator fatigue in digital nuclear power plants under emergency scenarios. However, due to the demanding nature of licensed nuclear operators, who must undertake shift monitoring and extensive routine training, it is challenging to recruit them for laboratory-based experiments. In accordance with standard practices in nuclear-related human factors research, this study recruited 23 healthy right-handed male college students (mean age: 19.70 ± 1.02 years; body mass: 70.83 ± 10.07 kg; height: 173.51 ± 5.96 cm; shoulder height: 146.52 ± 6.06 cm; leg length: 101.44 ± 5.15 cm; knee height: 51.57 ± 2.68 cm) through paid participation. All participants had no history of musculoskeletal disorders and reported sufficient sleep and no intake of substances affecting heart rate prior to the experiment. Their muscular endurance, physical fitness level, and autonomic nervous system responses are fundamentally different from those of professional licensed operators, which may restrict the external validity and direct generalization ability of the proposed fatigue monitoring model. Given that previous research indicates no significant difference in error rates between trained non-professionals and licensed operators when performing skill-based tasks, participants underwent a series of training sessions and assessments before the experiment to ensure they were proficient in the task, effectively simulating skilled personnel. The experiment was conducted in a controlled laboratory environment with a temperature of 18.37 ± 0.97 °C and relative humidity of 40.00 ± 9.82%. A simulated accident-handling scenario, modeled after a floating offshore nuclear power plant, was developed by the research team using Visual Studio and C++. As shown in Figure 3, the experimental interface consisted of four screens and a trackball-based human–machine interaction system, designed to investigate the characteristics of fatigue accumulation during monitoring operations performed by the operators.

This experiment draws upon methods commonly used in human factors engineering research under swing environments, wherein swing conditions are established by inputting predefined six-degree-of-freedom motion data. Additionally, it references the test conditions for surface vessels specified in the Guidelines for Environmental Testing and Engineering of Ship Equipment—Inclination and Swing. After comprehensive consideration, the swing conditions used in this study were determined as shown in Table 1.

Based on the results of on-site investigations, a simulated operational environment for digital industrial system main control room operators under swing conditions was constructed, as illustrated in Figure 3. The swing conditions were implemented using a YSL06-500 KG six-degree-of-freedom motion platform developed by YSL Technology. Signal acquisition was carried out using the MR 3.16 16-channel wireless surface electromyography (sEMG) system from Noraxon (USA) and the ErgoLAB human–environment synchronization acquisition system from Beijing Jingfa Technology Co., Ltd. Experimental consumables included LT–301 electrodes from Shanghai Litum Medical Equipment Co., Ltd. and medical pressure-sensitive adhesive tape provided by Hainuo Group.

### 3.2. Data Collection

This experiment investigates operator fatigue by focusing on key indicators of fatigue accumulation, incorporating surface electromyography (sEMG), heart rate variability (HRV), and subjective fatigue assessments. Under swing conditions, participants in digital industrial environments perform multi-screen monitoring tasks using an embedded trackball at a computer workstation. To maintain postural stability and ensure task execution amid ship-induced motion disturbances, the lumbar stabilizing muscles, particularly the erector spinae and multifidus, engage in prolonged isometric contractions. Concurrently, the upper trapezius and infraspinatus muscles coordinate with the erector spinae to facilitate complex shoulder and arm movements, while the flexor carpi radialis adjusts wrist flexion angles in real-time to maintain precise control of the trackball. Moreover, the upper trapezius is involved in regulating head posture by modulating muscle tension at a high frequency, supporting visual tracking across multiple display screens.

Given these biomechanical requirements, sEMG data were collected from nine muscle groups: the left and right upper trapezius, left and right infraspinatus, left and right erector spinae, left and right multifidus, and the right flexor carpi radialis. This configuration enabled analysis of localized muscle fatigue accumulation patterns. In addition, HRV signals were recorded to evaluate the autonomic nervous system’s responses, as swing conditions are likely to activate sympathetic activity and suppress parasympathetic function due to combined postural and cognitive demands. Subjective perceptions of fatigue were also captured using the Category Ratio-10 (CR-10) scale [41]. Although the Borg CR-10 scale was initially developed for assessing local physical exertion and dyspnea, it has been widely validated and adopted in recent studies to evaluate integrated cognitive-postural fatigue during prolonged operational tasks, especially under continuous postural control and mental load conditions. Its ordinal sensitivity to cumulative fatigue makes it suitable for the present study, where operators experience combined physical instability and cognitive demand. Comprehensive information regarding the variables involved is summarized in Table 2.

To ensure that the measured sEMG and HRV variations accurately represent operational fatigue rather than motion sickness, mental workload, or emotional stress, strict differentiation and control strategies were implemented. First, participants who reported dizziness, nausea, or motion sickness during the pre-experiment adaptation stage were excluded to avoid interference from acute motion-related discomfort. Second, the task difficulty, operation process, and cognitive demand were kept constant throughout the experiment to stabilize mental workload and prevent unexpected fluctuations. Third, typical physiological patterns were used for differentiation: operational fatigue shows a gradual decrease in sEMG median frequency and a sustained increase in HRV LF/HF ratio, whereas motion sickness and acute emotional stress mainly induce rapid heart rate elevation without obvious muscle fatigue characteristics. Finally, all participants maintained a stable emotional state without acute stress, which was verified by post-experiment subjective reports.

From a cognitive psychology perspective, humans naturally tend to perceive intensity in a “low–medium–high” tripartite framework. This categorization aligns with everyday linguistic habits for describing levels of experience (e.g., “weak,” “moderate,” “very strong”), allowing respondents to quickly self-assess their state and enabling researchers to interpret attitude strength in a hierarchical manner.

To justify the validity of the three-tier fatigue classification boundaries derived from the Borg CR-10 scale (0–3 normal, 4–6 fatigue, 7–10 severe fatigue), this study aligns the segmentation rule with classic human factor research on physical–cognitive combined fatigue [42]. The tripartite intensity partition conforms to the widely accepted psychological perception gradient of operator load reported in the existing literature. To confirm the stability of classification results under minor boundary adjustments, we retrospectively reclassify all existing samples with two alternative cutoff schemes without collecting new experimental data: scheme 1 (0–2 normal, 3–6 fatigue, 7–10 severe fatigue) and scheme 2 (0–3 normal, 5–7 fatigue, 8–10 severe fatigue). Reanalysis based on the original dataset indicates that the overall accuracy fluctuation of all four models stays within ±1.1%, and the relative performance ranking of the four algorithms remains unchanged. This retrospective reanalysis demonstrates that slight shifts to CR-10 thresholds do not alter the core research conclusions, confirming the robustness of the label division strategy.

From a data analysis standpoint, the tripartite classification simplifies statistical processing while preserving ordinal scale information. It avoids the pitfalls of overprecision that may arise from treating responses as continuous variables and offers clearer group-level comparisons than more granular categorizations, thereby enhancing both interpretability and practical utility of the results. Accordingly, the fatigue classification used in this study is presented in Table 3.

Due to observations during the experiment indicating that participants were unable to continue under high swing conditions beyond 80 min, the maximum task duration was set at 80 min. To fully capture temporal dependencies and cumulative fatigue effects, the whole task was divided into eight successive time intervals (10, 20, 30, 40, 50, 60, 70, and 80 min). sEMG and HRV features were extracted continuously at each time point to reflect the progressive development of fatigue. Both static features and time-related trend variations were included in the feature vector, enabling the model to learn temporal dependencies and cumulative changes in physiological signals during fatigue evolution. Considering that muscle fatigue is typically associated with a leftward shift and reduction in frequency in the sEMG power spectrum, and that the median frequency (MF) offers strong resistance to noise and low error in the frequency domain analysis of sEMG signals, MF was selected as the key indicator for evaluating cumulative muscle fatigue. The sEMG signals were digitized at a sampling rate of 1000 Hz. A fourth-order Butterworth bandpass filter with passband of 10–500 Hz was applied to retain the low-frequency spectral peak (5–15 Hz) corresponding to mean motor unit discharge frequency, alongside a narrow-stopband 50 Hz notch filter to remove powerline interference. Notch filtering near 50 Hz may introduce mild spectral distortion of sEMG frequency components related to fatigue features; to mitigate this limitation, we adopted a narrow stopband for the notch filter and implemented post-filter spectral interpolation to recover distorted frequency information around 50 Hz, following standardized sEMG denoising workflows for dynamic vibration environments [22]. The median frequency (MF) was calculated using a 250 ms sliding Hanning window with 50% overlap, and the FFT length was set to 128 points. Before formal experiments, Maximum Voluntary Contraction (MVC) of each target muscle was measured three times, and the average MVC value was used as the baseline to normalize sEMG amplitude features. This normalization procedure effectively reduces individual differences and ensures reliable data comparison across participants.

### 3.3. Experimental Procedure

A preliminary experiment was conducted to ensure that participants adapted well to the tasks and experimental conditions. Participants received training covering nuclear power principles, experimental interface operation, procedural execution, and task handling. They underwent repeated practice sessions in the laboratory, at least three times, until they reported full familiarity with the experimental process and achieved flawless performance in emergency handling tasks with stable system operation. Participants also adapted to two levels of swing conditions, with those experiencing dizziness or discomfort excluded. Additionally, they mastered trackball operations including screen scrolling, cursor positioning, and target clicking. During the formal experiment, the principal investigator first explained the procedure to the participants, who then familiarized themselves with the equipment and signed informed consent forms. Participants performed a 5-min aerobic warm-up guided by a video, followed by a seated rest period. The investigator prepared the participants by removing hair from nine muscle sites and cleaning the skin with alcohol wipes. Paired electrodes spaced 2 cm apart were placed longitudinally along the muscle fibers on the muscle belly. After cleaning the earlobe with alcohol, PPG (photoplethysmography) sensors for HRV acquisition were affixed there, and data cables were secured to the right upper arm with straps.

## 4. Model Construction

### 4.1. Data Partitioning

All data partitioning and cross-validation procedures strictly adopt subject-independent segmentation rules to avoid sample-level data leakage, which would artificially inflate classification accuracy and threaten the reliability of the experimental results. All samples belonging to a single participant are entirely allocated to either training folds or test folds; no individual subject’s data is split across training and test subsets.

Two subject-level partitioning schemes are clearly distinguished to resolve logical conflicts between stratified LOSO-CV and the 9:1 split.

1. Stratified Leave-One-Subject-Out Cross-Validation (LOSO-CV, primary evaluation standard): For the 23 recruited subjects, each cross-validation iteration retains all samples of one complete subject as the held-out test set, and the remaining 22 subjects constitute the training set. Stratified sampling within each fold maintains consistent normal/fatigue/severe-fatigue class ratios between training and test subsets.
2. Auxiliary 9:1 stratified subject group split (only for auxiliary robustness inspection, not the basis of core results): Participants are randomly grouped into a training cohort (90% of subjects) and a test cohort (10% of subjects) rather than splitting individual samples. All samples from selected subjects are kept intact in corresponding subsets, and stratified grouping ensures consistent global fatigue class proportions. This auxiliary split only serves as a supplementary inspection and is not adopted to generate the main quantitative results reported in this paper.

**Figure 4:**
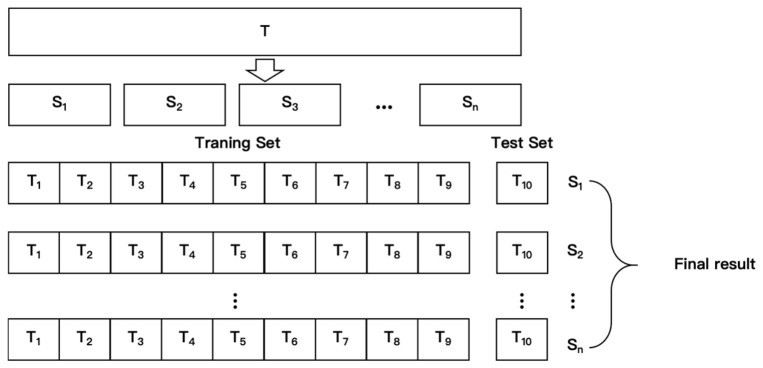
Stratified Leave-One-Subject-Out Cross-Validation flowchart.

Figure 4 illustrates the complete workflow of subject-independent stratified LOSO-CV, where all samples from one subject are treated as an indivisible unit during data segmentation.

### 4.2. Model Evaluation Index

To assess the model’s performance on this dataset, key evaluation metrics including accuracy (ACC), precision (P), recall (R), and F1-Score were selected. Furthermore, a confusion matrix was used to further elaborate on the model’s classification efficacy. The more the elements of the confusion matrix cluster along the diagonal, the more favorable the model’s training outcomes. The elements of the confusion matrix, true negative (TN), false negative (FN), false positive (FP), and true positive (TP), correspond to distinct classification results, as detailed in Table 4.

Accuracy represents the proportion of correctly classified samples out of all samples, with higher values indicating better model performance. The calculation formulas for Accuracy (ACC), Precision (P), Recall (R), and F1-Score are as follows [43,44,45,46,47,48]:

(12) $$ACC=\frac{TN+TP}{TN+FN+TP+FP}$$

(13) $$P=\frac{TP}{TP+FP}$$

(14) $$R=\frac{TP}{TP+FN}$$

(15) $$F1-Score=\frac{2\times P\times R}{P+R}$$

Additionally, this study employs the Matthews Correlation Coefficient (MCC), Receiver Operating Characteristic (ROC) curve area, and Precision–Recall Curve (PRC) to evaluate the model [49,50]. MCC is a performance metric specifically designed for binary classification models. It considers all four categories of the confusion matrix, true positives (TP), true negatives (TN), false positives (FP), and false negatives (FN), providing a comprehensive assessment of classification quality. The MCC value ranges from −1 to 1, with its formula defined as follows:

(16) $$MCC=\frac{TP\times TN-FP\times FN}{\sqrt{\left(TP+FP\right)\left(TP+FN\right)\left(TN+FP\right)\left(TN+FN\right)}}$$

The MCC ranges from −1 to 1, where a value of 1 indicates perfect classification, 0 reflects performance equivalent to random guessing, and −1 denotes a complete inverse correlation between predictions and actual outcomes. MCC is particularly well-suited for evaluating imbalanced classification problems, as it considers true and false positives and negatives across all classes, offering a balanced assessment regardless of class distribution. Compared to traditional evaluation metrics, MCC provides a more holistic measure of a model’s predictive quality. The ROC curve visualizes the relationship between the true positive rate (TPR) and the false positive rate (FPR) over a range of classification thresholds. It is a standard tool for assessing the discriminative ability of classification models. As the threshold varies, the ROC curve demonstrates how TPR and FPR shift, with an optimal curve approaching a TPR of 1 and an FPR of 0—indicating flawless performance. In contrast, the PRC depicts the trade-off between precision and recall and is especially informative when dealing with datasets exhibiting class imbalance, as it emphasizes the performance of the minority class.

### 4.3. Model Prediction Results

**Figure 5:**
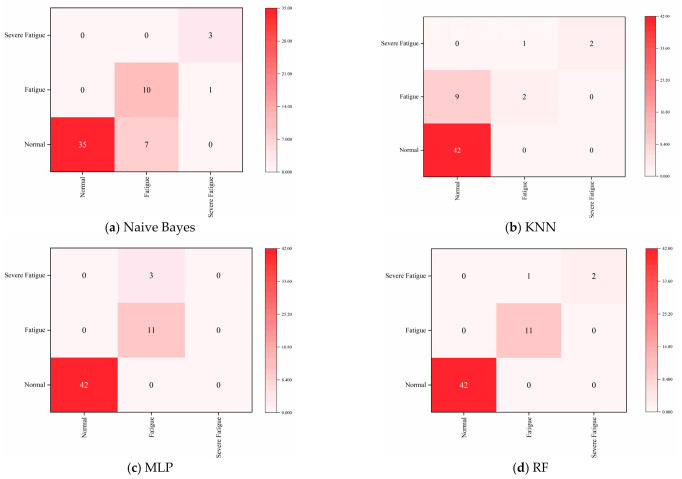
Testing set confusion matrices of the four algorithms.

The experimental software environment consists of the Windows 11 operating system, the Anaconda3 distribution, Python version 3.7, and CUDA 11.2. TensorFlow 2.9.0 is utilized as the primary deep learning framework. On the hardware side, the setup features an Intel(R) Core(TM) i7-14700KF processor operating at a base frequency of 3.40 GHz, along with a single NVIDIA GeForce RTX 3090 GPU equipped with 24 GB of dedicated memory. Using the TensorFlow framework, four machine learning models were implemented based on the collected datasets. The results derived from these models are summarized in Figure 5 and detailed in Table 5, Table 6, Table 7 and Table 8. The specific hyperparameters of each machine learning model are set as follows. For Naive Bayes, a Gaussian naive Bayes classifier was used with default parameters. For KNN, the number of neighbors was k = 5, and Euclidean distance was used as the distance metric. For MLP, the network consists of an input layer, two hidden layers (128 and 64 neurons, respectively), and an output layer; the sigmoid activation function was adopted; the Adam optimizer was used with a learning rate of 0.001; batch size was 32, and the training epoch was 100. For Random Forest, the number of decision trees was n_estimators = 500, max_depth = 15, min_samples_split = 2, and the Gini index was used as the splitting criterion with bootstrap sampling enabled.

Overall, the Random Forest (RF) model delivered the strongest classification performance, achieving an accuracy of 98.2% by correctly classifying 56 samples with only 1 misclassification. This highlights a high degree of consistency between the predicted and actual labels. The Multilayer Perceptron (MLP) ranked second with an accuracy of 94.6%, yet its misclassifications were heavily skewed toward the severe fatigue category, where 3 out of its 3 misclassifications occurred. Naive Bayes and K-Nearest Neighbors (KNN) showed relatively weaker performance, with accuracies of 85.7% and 82.1%, respectively. These findings confirm the superiority of ensemble learning methods like RF in multimodal fatigue recognition. By aggregating multiple decision trees, RF reduces variance, thereby enhancing robustness against the complex features encountered under swing conditions.

In terms of state recognition, RF and MLP displayed distinct strengths across different categories. For the normal state, KNN and RF and MLP achieved a perfect true positive rate (TPR) of 100%, indicating exceptional feature extraction capabilities for normal conditions. However, RF maintained a balanced precision and recall, while MLP completely misclassified all severe fatigue samples, exposing its weakness in detecting extreme states. For the fatigue state, RF attained a TPR of 1.000, a precision of 0.917, and an F1-Score of 0.957, reflecting excellent overall performance. Although MLP achieved a TPR of 1.000 for the fatigue state, its precision dropped to 0.786, pointing to false positives where normal states were incorrectly identified as fatigue. In identifying severe fatigue, RF achieved a TPR of 0.667 with a precision of 1.000, significantly outperforming Naive Bayes and KNN. This demonstrates high classification reliability even with the limited sample size. MLP, on the other hand, had a TPR of 0 for severe fatigue, likely due to overfitting caused by the low number of severe fatigue samples, resulting in insufficient learning of features for this class.

Analysis of the confusion matrix revealed that misclassifications were primarily concentrated in specific scenarios. Naive Bayes, which relies on the assumption of feature independence, was hindered by the strong correlations between sEMG and HRV signals under swing conditions, leading to 7 normal samples being misclassified as fatigue. KNN’s limitations in distance metrics within high-dimensional spaces, compounded by noise from swing-induced feature disturbances, reduced the representativeness of neighbors; among its 10 misclassifications, 9 fatigue samples were misclassified as normal, and 1 severe fatigue samples were misclassified as fatigue. MLP suffered from gradient vanishing issues due to hidden layer designs such as sigmoid activation functions, impairing the extraction of nonlinear features in deeper layers; of its 3 misclassifications, all were misses of severe fatigue. RF’s main confusion occurred between fatigue and severe fatigue states, accounting for 1 out of its 1 misclassifications. This may be because physiological indicator differences are masked by noise under swing conditions—for instance, the LF/HF ratio of HRV fluctuates more intensely under high load, blurring class boundaries.

In the context of imbalanced samples (this study collected a total of 558 valid samples, including 24 cases of severe fatigue, accounting for 4.3%), the RF model demonstrated balanced and excellent performance, supported by a superior Matthews Correlation Coefficient (MCC = 0.979), ROC curve area (1.000), and PRC area (1.000). This robustness makes RF highly valuable for industrial safety monitoring, as its false positive rate for severe fatigue alarms was extremely low (FP RATE = 0.004), effectively minimizing production disruptions caused by false alarms. In contrast, while MLP performed well for normal and fatigue states, its failure to detect severe fatigue poses potential safety risks. Naive Bayes and KNN’s high misclassification rates make them unsuitable for meeting stringent real-time monitoring requirements.

Among all physiological features, sEMG median frequency (MF) and HRV LF/HF ratio contributed most significantly to fatigue classification. The gradual decline of sEMG MF indicates reduced muscle fiber conduction velocity caused by cumulative muscle fatigue, while the elevated LF/HF ratio reflects sympathetic activation and autonomic imbalance under continuous postural and cognitive load. Both features possess clear and well-established physiological relevance to operational fatigue in dynamic swing environments.

Multimodal fusion of sEMG and HRV significantly improved classification accuracy and robustness compared with single-signal methods. The sEMG-only model was vulnerable to motion artifacts, whereas the HRV-only model lacked information about physical fatigue. The fused framework compensated complementary information from muscle activity and autonomic response, leading to consistent performance improvements across all evaluation metrics.

For practical application concerns, standardized electrode placement, fixed adhesive patches, and elastic straps were used to reduce sensor displacement and positioning errors. Solid gel electrodes were adopted to alleviate interference from sweating and motion-induced changes in skin impedance during prolonged operation. The adaptive filtering and artifact rejection module further ensured signal stability, making the system suitable for long-term industrial monitoring.

The study has limitations, including a small number of severe fatigue samples (only 24). Future work can address this by extending trial durations or recruiting more participants to enrich the dataset for extreme states. Currently, only sEMG, HRV, and subjective ratings were fused; incorporating additional modalities such as eye-tracking and facial expression analysis could enhance feature dimensionality. The RF model’s computational complexity, with 500 decision trees, requires the application of model compression techniques like pruning or quantization to make it suitable for industrial edge computing devices. Future research could explore integrating Transformer architectures with RF, leveraging attention mechanisms to better capture key fatigue features such as frequency domain changes in erector spinae sEMG signals, while introducing transfer learning to address small-sample challenges. This would advance the model’s engineering application in complex industrial scenarios such as offshore floating nuclear power plants. Several limitations should be noted. First, all 23 participants were young male college students without professional industrial or nuclear operation experience. This demographic differs from licensed industrial operators in age, physical conditioning, muscle endurance, and long-term operational experience, which may restrict the external validity of the proposed fatigue monitoring model. Future studies will recruit licensed operators with professional backgrounds, include female participants, and expand the age range to improve generalization.

## 5. Conclusions

This study presents a real-time fatigue monitoring framework for industrial operators under swing conditions, using sEMG and HRV multimodal sensors combined with machine learning. A simulated emergency operation experiment was carried out on a six-degree-of-freedom platform with three swing levels, and fatigue classification was implemented using four typical algorithms. The results demonstrate that the Random Forest model achieves the optimal performance, with an overall accuracy of 98.2%, 100% true positive rate for normal state and fatigue, and 66.7% precision for severe fatigue. The proposed multimodal fusion method effectively reduces the interference of swing motion artifacts and improves the robustness and accuracy of fatigue recognition in dynamic industrial environments. Complementary physiological information from sEMG and HRV jointly suppress swing noise; subject-level stratified LOSO-CV eliminates sample leakage risks; imbalance mitigation strategies and per-class uncertainty quantification address the scarcity of severe-fatigue samples; and retrospective cutoff sensitivity analysis verifies the stability of Borg CR-10 fatigue labeling. Bootstrap confidence intervals and pairwise significance tests statistically validate the outstanding classification ability of Random Forest. Although stratified cross-validation was used to alleviate class imbalance, the limited number of severe fatigue samples (24 samples) may still affect the recognition stability of this category. Future studies will extend the experimental duration and recruit more participants to enrich the extreme fatigue dataset. Also, only conventional machine learning methods were used in this study to validate the proposed multimodal fusion strategy with simplicity and real-time efficiency. Advanced temporal deep learning models such as LSTM, CNN-LSTM, and Transformer will be systematically investigated in future work to enhance the modeling of sequential physiological signals.

This sensor-based intelligent monitoring method provides a reliable technical solution for real-time fatigue detection of operators in high-risk digital industrial systems, such as offshore floating nuclear power plants. It supports timely intervention for operational fatigue, reduces the probability of human-caused accidents, and helps improve the safety, personnel reliability, and long-term sustainability of critical industrial operations. Future work will focus on expanding the dataset of extreme fatigue states to narrow the confidence interval of severe-fatigue classification, integrating more behavioral and physiological sensors, optimizing the model for edge computing deployment, recruiting professional operators to improve external validity, and exploring advanced imbalanced learning algorithms to boost detection performance for safety-critical severe fatigue.

## Figures and Tables

**Table 1:** Vibrating conditions.

Degree of Freedom	Static		Low		High	
	Amplitude (mm)/Angle (°)	Time Period/s	Amplitude (mm)/Angle (°)	Time Period/s	Amplitude (mm)/Angle (°)	Time Period/s
Longitudinal oscillation	±0	0	±45	10	±90	10
Lateral oscillation	±0	0	±45	5	±90	5
Vertical oscillation	±0	0	±45	10	±90	10
Lateral swing	±0	0	±9	5	±18	5
Longitudinal swing	±0	0	±5	10	±10	10
Vertical swing	±0	0	±2.5	10	±5	10

**Table 2:** Borg’s CR-10 scale.

Score	Description of Fatigue Sensation
10	Extremely Strong
9	
8	
7	Very Strong
6	
5	Strong
4	
3	Moderate
2	Weak
1	Extremely Weak
0	Nothing at all

**Table 3:** Fatigue Category Classification Table.

Score	Fatigue Categories
**0–3**	**Normal**
**4–6**	**Fatigue**
**7–10**	**Severe Fatigue**

**Table 4:** Classification of the situation of the model prediction results.

Classification	Category A	Category B
Predicted as Category A	TN	FN
Predicted as Category B	FP	TP

**Table 5:** The test evaluation results for Naive Bayes.

Category	TP Rate	FP Rate	Precision	Recall	F1-Score	MCC	ROC Area	PRC Area
Normal	0.833	0.000	1.000	0.833	0.909	0.745	0.990	0.997
Fatigue	0.909	0.156	0.588	0.909	0.714	0.651	0.885	0.802
Severe Fatigue	1.000	0.019	0.750	1.000	0.857	0.858	0.987	0.806
Overall Evaluation	0.857	0.032	0.906	0.857	0.868	0.733	0.969	0.948

**Table 6:** The test evaluation results for KNN.

Category	TP Rate	FP Rate	Precision	Recall	F1-Score	MCC	ROC Area	PRC Area
Normal	1.000	0.643	0.824	1.000	0.903	0.542	0.927	0.967
Fatigue	0.182	0.022	0.667	0.182	0.286	0.282	0.865	0.497
Severe Fatigue	0.667	0.000	1.000	0.667	0.800	0.809	0.833	0.685
Overall Evaluation	0.821	0.487	0.802	0.821	0.776	0.505	0.910	0.859

**Table 7:** The test evaluation results for MLP.

Category	TP Rate	FP Rate	Precision	Recall	F1-Score	MCC	ROC Area	PRC Area
Normal	1.000	0.000	1.000	1.000	1.000	1.000	1.000	1.000
Fatigue	1.000	0.067	0.786	1.000	0.880	0.856	1.000	1.000
Severe Fatigue	0.000	0.000	0.000	0.000	0.000	0.000	1.000	0.997
Overall Evaluation	0.946	0.013	0.904	0.946	0.923	0.918	1.000	1.000

**Table 8:** The test evaluation results for RF.

Category	TP Rate	FP Rate	Precision	Recall	F1-Score	MCC	ROC Area	PRC Area
Normal	1.000	0.000	1.000	1.000	1.000	1.000	1.000	1.000
Fatigue	1.000	0.022	0.917	1.000	0.957	0.947	1.000	1.000
Severe Fatigue	0.667	0.000	1.000	0.667	0.800	0.809	1.000	1.000
Overall Evaluation	0.982	0.004	0.982	0.982	0.981	0.979	1.000	1.000

## Data Availability

The data that support the findings of this study are available from the corresponding author upon reasonable request.
